# Variation in Young Driver Training Requirements by State

**DOI:** 10.1001/jamanetworkopen.2024.17551

**Published:** 2024-06-17

**Authors:** Elizabeth A. Walshe, Daniel Romer, Nina Aagaard, Flaura K. Winston

**Affiliations:** 1 The Center for injury Research and Prevention, Children’s Hospital of Philadelphia, Pennsylvania; 2 The Annenberg Public Policy Center, University of Pennsylvania, Philadelphia

## Abstract

This cross-sectional study evaluates graduated driver licensing laws and requirements for behind-the-wheel training or adult-supervised practice for drivers younger than 18 years.

## Introduction

Graduated driver licensing laws (GDL) are the national strategy to address young driver motor vehicle crashes, which are a leading cause of injury and mortality for US teens.^1^ The basic tenets of GDL are to delay full licensure until older ages and impose driving restrictions under the intermediate license. However, despite GDL, crash rates remain high.

Prior studies^2^ have quantified that the majority (75%) of young novice driver crashes are due to driver errors, particularly related to detecting and responding to hazards. Interventions that aim to improve these crash avoidance skills before licensure should help address this preventable public health crisis.

Some states have supplemented GDL with training requirements for adult-supervised practice hours (ASP) and/or professional behind-the-wheel training (BTW). Where both are required before licensure, such as in Ohio for drivers younger than 18 years, lower crash rates have been observed early in licensure, compared with new drivers aged 18 years who did not complete BTW training.^3^ States without training mandates or that rely on ASP alone may be missing an opportunity to enhance safety-critical driving skills before licensure. We undertook a contemporary review of license policies across the 50 states to determine BTW and ASP requirements before intermediate licensure for those younger than 18 years and whether these mandates are truly required (or can be replaced).

## Method

From May to August 2022, we catalogued each state’s requirements for intermediate and/or junior licensure through online information and telephone inquiries. This study did not require institutional review board oversight or informed consent at the Children's Hospital of Philadelphia because it did not meet the requirement for human participants research. The study followed Strengthening the Reporting of Observational Studies in Epidemiology (STROBE) reporting guidelines. Flow diagrams were created for the process and requirements for licensure in each state. States were categorized as: (1) requiring ASP only if no BTW was required and (2) requiring BTW only if BTW could not be replaced with ASP. Data were analyzed from July to December 2022.

## Results

Most states (45 [90%]) required a driver to be at least 16 years of age to obtain an intermediate license. As shown in the Figure, the majority of states (44 [88%]) required some form of either ASP and/or BTW driving. A total of 28 (56%) required both BTW and ASP, 3 (6%) required ASP and allowed for replacement of BTW, 11 (22%) required ASP only, and another 6 (12%) required neither. Two (4%) states required BTW only for those younger than 16 years.

**Figure.**
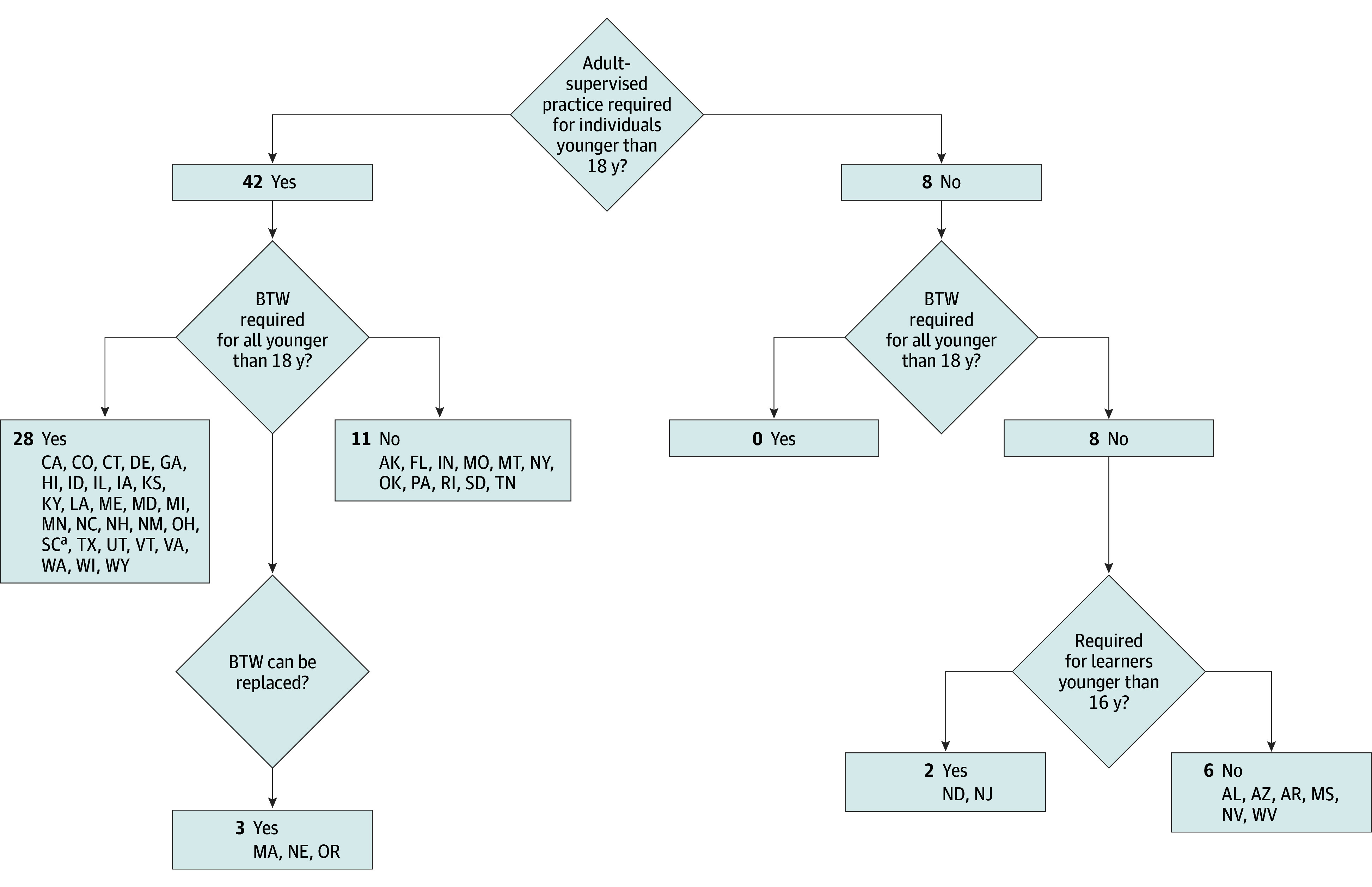
Behind the Wheel Training (BTW) and Adult Supervised Practice Requirements for Intermediate License Across the 50 US States ^a^SC requires adult supervised practice for all learners aged less than 17 years, but not for learners aged 17 years.

## Discussion

In this study, the majority of states (28) required both BTW training and ASP. However, 17 states had no BTW requirements, 6 of which had no ASP requirement either. Three additional states encouraged BTW but allowed replacement with ASP.

Despite the potential benefits of BTW, it can create potential disparities in the ability to drive legally; training is costly and favors those with access to sufficient finances.^4^ Newer online training has the potential to both enhance BTW or ASP while also improving access to quality training in critical safety skills.^5^ Given greater internet accessibility, online training programs could be a strategy to increase access to training and reduce disparities in licensure and crashes. Randomized clinical trials of driver training are needed to determine their relative efficacy.

This study has limitations. License policies may have been updated since this review in 2022. In addition, because of variations in crash reporting by state, direct comparisons across states preclude the ability to examine variations in training requirements on crash outcomes.

## Conclusions

In all states, clinicians should be aware that although their patients may be licensed, they may be ill prepared for safe driving. Due to individual variability in cognitive development and crash risk among young drivers, clinicians should generally advise parents to go beyond state minimum requirements as needed for their child. In particular, teens with medical or other conditions that could impact crash risk^6^ may benefit from training beyond what is provided by supervised practice alone.

## Notes

Supplement.
Data Sharing Statement
