# Errors in Results and Figure

**DOI:** 10.1001/jamanetworkopen.2024.32848

**Published:** 2024-08-20

In the Research Letter titled “Variation in Young Driver Training Requirements by State,”^1^ published June 17, 2024, there were errors in the Results and Figure. Three states were miscategorized as requiring behind-the-wheel training for all individuals aged less than 18 years; 2 states require training only for learners aged less than 16 years, and 1 state allows for replacement of training with adult supervised practice. This article has been corrected.^1^
