# Supplementary material for: Variation in Young Driver Training Requirements by State
Source: JAMA Netw Open. 2024 Jun 17;7(6):e2417551. doi: 10.1001/jamanetworkopen.2024.17551 (PMC11184456; doi:10.1001/jamanetworkopen.2024.17551)
Supplement: Supplement. — Data Sharing Statement [file jamanetwopen-e2417551-s001.pdf]

Data Sharing Statement

Walshe. Variation in Young Driver Training Requirements by State. *JAMA Netw Open*.
Published June 17, 2024. doi:10.1001/jamanetworkopen.2024.17551

Data
Data available: Yes
Data types: Data (not involving human participants)
How to access data: Corresponding author email walshee@chop.edu
When available: With publication

Supporting Documents
Document types: None

Additional Information
Who can access the data: Researchers whose proposed use of the data has been approved.
Types of analyses: Research and policy review.
Mechanisms of data availability: With an agreement.
